# The Relationship Between Neutrophil Extracellular Traps and CD8^+^ T Lymphocytes in Cancer: A Comprehensive Review of Current Data

**DOI:** 10.3390/cancers18132059

**Published:** 2026-06-25

**Authors:** Kellyn E. McKee, Hongji Zhang, Allan Tsung, Samantha M. Ruff

**Affiliations:** 1 University of Virginia School of Medicine, Charlottesville, VA 22903, USA; 2 Department of Surgery, University of Virginia Health, Charlottesville, VA 22903, USA; jhn5wx@virginia.edu (H.Z.); crf9aa@uvahealth.org (A.T.); jzp9rg@uvahealth.org (S.M.R.)

**Keywords:** cancer, neutrophils, neutrophil extracellular traps, NETosis, CD8^+^ T cells, immune evasion, immunotherapy

## Abstract

Neutrophil extracellular traps (NETs) are web-like structures composed of decondensed DNA, histones, and proteins released by activated neutrophils. Originally identified as an innate defense mechanism against pathogens, NETs have since been implicated in cancer progression and immune evasion. Within the tumor microenvironment (TME), NETs suppress anti-tumor immunity through multiple mechanisms, including the physical exclusion of CD8^+^ cytotoxic T lymphocytes from the tumor interior and upregulation of exhaustion markers via checkpoint ligands. This review synthesizes current preclinical and clinical evidence on the interplay between NETs and CD8^+^ T cells across multiple malignancies, including non-small cell lung cancer, pancreatic ductal adenocarcinoma, cholangiocarcinoma, colorectal cancer, bladder cancer, hepatocellular carcinoma, skin cancer, and penile cancer. Cancer-specific mechanisms of NET-mediated immune suppression are discussed, including IL-8, IL-17, CXCL6, and TGF-β-driven NETosis pathways. Clinical data consistently demonstrate that elevated NET levels correlate with reduced CD8^+^ T cell infiltration, T cell dysfunction, and worse patient outcomes. Emerging therapeutic strategies targeting this axis are reviewed, including DNase I-mediated NET degradation, Peptidyl arginine deiminase 4 (PAD4) inhibition, CXCR2 blockade, and combination approaches with immune checkpoint inhibitors. These interventions have shown promise in restoring CD8^+^ T cell cytotoxicity and overcoming immunotherapy resistance in preclinical models. Collectively, the evidence supports the NET-CD8^+^ T cell axis as a promising prognostic and therapeutic target warranting further clinical investigation.

## Simple Summary

Cancer cells can evade the immune system by taking advantage of a natural defense mechanism used by white blood cells called neutrophils. When activated, neutrophils release web-like structures made of DNA and proteins, known as extracellular traps. These extracellular traps normally help fight infections, but in cancer, they can instead protect tumors from immune cells that would otherwise destroy them. They do this by forming physical barriers around tumors and releasing signals that weaken and deactivate these protective immune cells. This review brings together the latest research on how these neutrophil extracellular traps interact with cancer-fighting immune cells across multiple cancer types, including lung, pancreatic, liver, colorectal, bladder, skin, and penile cancers. By understanding these interactions, researchers may be able to better predict patient outcomes and develop therapies that break down these traps, potentially restoring the immune system’s ability to fight cancer effectively.

## 1. Introduction

A functioning immune system relies on a delicate balance of feedback loops, inflammatory signaling, and anti-inflammatory checkpoints involving both innate and adaptive immune cells. While transient shifts favoring inflammation are beneficial in many acute illnesses, prolonged downregulation of anti-inflammatory cell types or bypassing of immune checkpoints contributes to pathologic conditions, including malignancy. Cancer progression is influenced by the complex interplay of cytokines and immune cells within the TME. As the primary effector of anti-tumor immunity, CD8^+^ cytotoxic T lymphocytes are central to this balance. However, recent data suggest that the efficacy of their adaptive response is heavily modulated by the innate immune landscape, specifically neutrophils and the extracellular traps they release. Although NETs have been shown to interact with a variety of adaptive immune populations, including CD4^+^ T cells, regulatory T cells, and B cells, their effects on CD8^+^ T cells are among the most extensively characterized and are particularly relevant to tumor progression, patient prognosis, and response to immunotherapy. Consequently, understanding the mechanisms by which NETs influence CD8^+^ T cells may provide unique insight into both cancer immune evasion and therapeutic resistance. The goal of this review is to synthesize the most up-to-date data regarding the relationship between NETs and CD8^+^ T cells in the TME and evaluate the potential for targeting this axis in cancer prognosis and treatment.

### 1.1. The Role of Neutrophils in Inflammation and NET Formation

Neutrophils are the most abundant leukocyte in human circulation and are traditionally characterized as short-lived first responders of the innate immune system [1]. They are involved in phagocytosis and clearance of invading pathogens through the release of reactive oxygen species and proteases. Beyond these bactericidal roles, neutrophils are now recognized as important immunomodulators [2]. By secreting a diverse array of cytokines and chemokines, they facilitate the recruitment and differentiation of macrophages, dendritic cells, natural killer cells, and both B and T lymphocytes [3]. Neutrophils can exhibit significant plasticity, commonly categorized into the N1 (anti-tumor) and N2 (pro-tumor) phenotypes. N1 neutrophils promote CD8+ T cell recruitment and tumor lysis while the TGF-β-induced N2 phenotype suppresses CD8+ T cell activity [4].

A defining feature of neutrophil activation is the release of NETs, which are web-like scaffolds composed of decondensed DNA, histones, and antimicrobial proteins from the neutrophil cytoplasm [5]. Initially identified as a defense mechanism to trap and kill extracellular pathogens, NETs have since been implicated in the pathogenesis of multiple disease states and malignancies [2].

**Figure 1:**
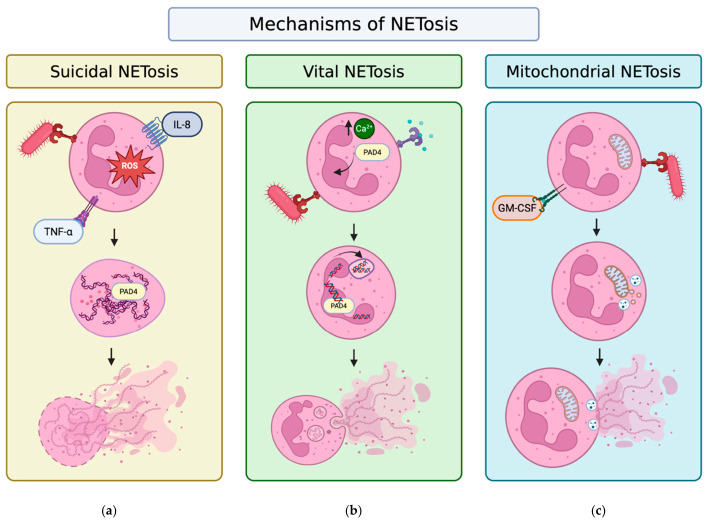
Neutrophils can generate NETs through three primary pathways. (**a**) In suicidal NETosis, neutrophil activation by bacteria or inflammatory cytokines leads to production of ROS via NADPH oxidase, triggering PAD4-mediated histone citrullination, chromatin decondensation, and breakdown of the nuclear envelope. This causes plasma membrane rupture and cell death. (**b**) In contrast, vital NETosis is a process in which neutrophils expel vesicles containing segments of decondensed chromatin without compromising nuclear integrity. This pathway is initiated by stimuli such as microbes or complement proteins and depends on calcium-driven PAD4 activation, allowing NET release while preserving cellular functions. (**c**) Lastly, mitochondrial NETosis is a non-lytic pathway in which neutrophils selectively release mitochondrial DNA in response to stimuli such as GM-CSF, enabling NET formation without compromising cell survival. There is some ongoing controversy about how distinct these pathways truly are, and further investigation is needed to clarify the overlap and interplay between these NETosis mechanisms. Created in BioRender. Ruff, S. (https://BioRender.com/oqzkok4, accessed on 22 June 2026) is licensed under CC BY 4.0.

The formation of these structures, termed NETosis, occurs through at least three described pathways (Figure 1). The first pathway is termed suicidal NETosis, which involves NADPH oxidase-dependent disintegration of the neutrophil nuclear membrane, leading to cell death and expulsion of genomic DNA [6]. The second mechanism is vital NETosis, in which stimuli such as activated platelets, immune complexes, and granulocyte–macrophage colony-stimulating factor (GM-CSF) trigger PAD4-mediated histone citrullination, which decondenses segments of nuclear chromatin [7]. This important role of PAD4 was confirmed by experiments demonstrating that neutrophils from PAD4 knockout mice can migrate to sites of inflammation, but cannot release NETs [8]. Following decondensation, portions of DNA are then cleaved by proteases, sequestered into vesicles, and released without compromising nuclear membrane integrity, which enables the neutrophils to maintain some effector functions, such as phagocytosis [7]. The final mechanism is mitochondrial NETosis, in which specific inflammatory markers like C5a or lipopolysaccharide trigger mitochondrial DNA release [9]. Similarly to vital NETosis, this pathway does not induce cell lysis or render neutrophils nonfunctional, but rather induces microtubule and microfilament rearrangements that facilitate selective release of mitochondrial DNA [9].

However, the conflicting literature suggests that these pathways are not strictly isolated, separate entities, but rather exhibit some mechanistic overlap. Given the complexity of the pathways inducing vital and suicidal NETosis and questions regarding the true viability of neutrophils that have lost some of their nuclear DNA, more investigation is needed to elucidate whether vital and suicidal NETosis are distinct or exist along a shared spectrum [10]. Furthermore, the classification of mitochondrial NETosis remains a subject of ongoing debate and controversy within the field. Critics point to the scarcity of robust in vivo confirmation and argue that the selective expulsion of mitochondrial DNA may occasionally represent an experimental artifact or an overlapping hallmark of early apoptosis or necrosis, rather than a truly distinct, non-lytic programmed cell death pathway [10]. Consequently, establishing a universally accepted taxonomy for context-dependent NET release remains an active challenge.

### 1.2. NETs as Modulators of Innate and Adaptive Immunity in Cancer

NETs impact both the innate and adaptive immune system’s response to cancer cells. When encountered by innate immune cells, NETs can inhibit their ability to kill cancer cells through biochemical signaling and recruitment of inhibitory growth factors [11]. Pro-inflammatory macrophages are ordinarily responsible for mitigating these effects by enzymatically degrading NETs, but a feedback loop can emerge in which macrophage cell death and NET formation continuously amplify one another and impair the normal immune response [9].

In the adaptive immune system, NETs can prevent CD8^+^ T-cell-mediated killing through several mechanisms. First, NETs are reservoirs for inhibitory molecules like programmed death ligand 1 (PD-L1) and arginase 1 [12]. NET-bound PD-L1 binds PD-1 expressed on effector T cells, actively disrupting downstream T cell receptor signaling cascades and halting the production of effector cytokines like IFN-γ [12]. NET-associated arginase-1 has also been found to deplete local L-arginine from the extracellular microenvironment, triggering a state of metabolic starvation that stalls T cell proliferation [13]. This biochemical suppression is further compounded by the physical presence of the dense DNA meshwork itself, which can sterically hinder proper T cell receptor clustering and orientation within the TME [12]. There are additional disease-specific mechanisms by which CD8^+^ T cells and NETs interact that will be described in greater detail below.

### 1.3. Mechanisms of Tumor-Induced NETosis

NETs have been found in human tissue samples from at least 10 distinct cancer types, including breast, colorectal, and lung cancer [14]. Not only are NETs present in the TME, but cytokines produced by malignant cells can induce NETosis to promote immune evasion [15]. For example, tumor-derived IL-8 is one driver of NETosis in multiple cancer types. Experimentally, IL-8 alone is sufficient to trigger NETosis in human neutrophils from both healthy donors and cancer patients [14]. Tumors also produce high levels of IL-1, which induces production of IL-8 that can bind to CXCR1 and CXCR2 on neutrophils to induce NETosis [16]. However, blocking IL-8 does not stop all NET formation, which suggests other signaling molecules or environmental factors also contribute [14]. Granulocyte colony-stimulating factor (G-CSF) and high mobility group box 1 (HMGB1) have been established as additional mediators of tumor-driven NETosis. Malignancy-derived G-CSF accelerates systemic granulopoiesis and primes circulating neutrophils for NET release by promoting histone citrullination, which facilitates DNA unraveling [17]. The importance of G-CSF in NETosis was further supported by decreased NET production in neutrophils treated with anti-G-CSF antibodies [17]. HMGB1, which is a highly conserved damage-associated molecular pattern secreted within the hypoxic, necrotic zones of expanding tumors, has also been shown to increase histone citrullination via interactions with toll-like receptor 4 on neutrophils [18]. Neutrophils treated with HGMB1 have significantly greater chromatin decondensation and DNA release, while mice treated with anti-HMGB1 antibodies demonstrate decreased histone citrullination [18].

### 1.4. Current Controversies and the Methodological Challenges

It is important to preface this discussion of NET research with an acknowledgement that although understanding of NET biology is rapidly advancing, several important controversies and methodological challenges remain. There is no universally accepted set of markers that definitively distinguish NET formation from other processes involving extracellular DNA release, such as necrosis, apoptosis, or other forms of lytic cell death. As a result, experimental studies often rely on combinations of imaging, citrullinated histone detection, and DNA localization assays, which may vary in sensitivity and specificity. In addition, heterogeneity in experimental conditions, disease models, and stimulation protocols has contributed to inconsistent findings regarding NET formation and function. Mechanistically, NETosis itself is increasingly recognized as context-dependent, with both NADPH oxidase-dependent and independent pathways described, and emerging evidence suggesting that NET composition and functional effects may vary across tumor types and stages of disease. These challenges highlight the need for careful interpretation of NET-related data, particularly in complex settings such as the tumor microenvironment.

## 2. NETs and CD8^+^ T Cells

CD8^+^ T cells are adaptive effector lymphocytes that kill infected or malignant cells through the targeted release of cytokines and proteases [19]. To accurately evaluate how NETs impair these cells, it is important to distinguish between three distinct states of compromised anti-tumor immunity: T cell exclusion, T cell dysfunction, and T cell exhaustion. T cell exclusion is a spatial phenomenon wherein effector lymphocytes are physically barred from entering the tumor parenchyma. T cell dysfunction describes a broad state of diminished metabolic or effector capacity driven by local biochemical alterations in the microenvironment. Lastly, T cell exhaustion is a specific, progressive differentiation program marked by the sustained, high expression of inhibitory receptors, such as PD-1, TIM-3, and LAG-3. NETs induce one (or multiple) of these three states to impair CD8^+^ T lymphocyte cytotoxicity through two primary pathways: creation of a physical barrier and modulation of cytokine and chemokine signaling.

### 2.1. Physical Barrier

NETs can form a protective layer at the tumor-stroma interface that impairs immune cell infiltration of the tumor [9]. This physical barrier concept has been verified using multiple imaging modalities. Direct visualization of the tumor landscape using intravital microscopy of subcutaneous and liver tumors in mice demonstrated a predominance of NETs at the tumor periphery and decreased cytotoxic cell permeation into the tumor interior when NETs were present [16]. Multiplex immunofluorescence imaging of multiple human tissues from multiple cancer types, including NSCLC, bladder cancer, and metastatic melanoma, has also demonstrated an inverse relationship between NET density and CD8^+^ T cell density within tumors, reinforcing the idea that NETs act in part by creating a structural blockade preventing immune invasion [14]. Analysis of murine pancreatic tumor tissue using mass cytometry and multiplex immunofluorescence also demonstrated that IL-17-recruited neutrophils form NETs that exclude cytotoxic CD8+ T cells from tumors [20]. Importantly, anti-IL-17 therapy improved the spatial redistribution of CD8+ T cells, bringing them closer to tumor cells [20]. Because CD8^+^ T cells require direct stimulation by major histocompatibility complex I (MHC I) and costimulatory receptors, this cloaking effect impairs their activation and ability to kill tumor cells [8]. This effect was proven to be at least partially NET-dependent through the utilization of DNase I to break down the DNA component of the NET shield and/or PAD4 inhibitors to prevent NET formation. In both of these scenarios, CD8^+^ T cell tumor toxicity could be restored [8].

### 2.2. Cytokine and Chemokine Signaling

NETs contain high levels of the serine proteases neutrophil elastase and cathepsin G [21]. These NET-associated proteases can lead to poor immune infiltration of the TME by proteolytically degrading CD8^+^ T cell chemoattractants like CXCL9, CXCL10, and CXCL11 [22]. NETs also frequently contain PD-L1 and arginase-1, which can trigger the PD-1 signaling pathway in T cells. This leads to metabolic dysfunction and the upregulation of the exhaustion markers PD-1, TIM-3, LAG-3, which hinders CD8^+^ T cell cytotoxicity [23].

## 3. Cancer-Specific NET/CD8^+^ T Cell Interactions

### 3.1. Non-Small Cell Lung Cancer (NSCLC)

#### 3.1.1. Preclinical Data

Immunofluorescence staining of neutrophils from healthy donors compared to patients with NSCLC demonstrates markedly different NET profiles. Neutrophils from NSCLC patients produce more NETs and have a higher capacity for NET formation when stimulated [24]. This difference could potentially be mediated by A2AR signaling. In vitro experiments culturing macrophages with NSCLC cells demonstrate activation of A2AR mediates CXCL5 expression on macrophages, which can directly stimulate NETosis by binding CXCR2 on neutrophils [25]. CD8^+^ T cells co-cultured with these NETs demonstrated increased exhaustion markers and activation of the cGAS-STING signaling pathway, which further contributes to T cell dysfunction and decreases production of effector cytokines like IFN-γ and TNF-α [25]. Co-culture of NETs and NSCLC cells also upregulates mesenchymal markers such as vimentin and N-cadherin and downregulates epithelial markers (e.g., E-cadherin), which suggests NETs may play a role in the epithelial to mesenchymal transition [24]. This shift promotes metastasis by decreasing cancer cell adhesion, which is linked to a worse prognosis in NSCLC [26].

While this A2AR-CXCL5-CXCR2 axis appears to represent a unique mechanism of NETosis in NSCLC preclinical models, other pathways impact neutrophil profiles and NET release in the TME. Activation of TGF-β1/Smad3 signaling has been shown to increase the ratio of pro-tumor N2 neutrophils in tumor tissue from NSCLC patients [27]. The N2 neutrophil phenotype is associated with enhanced PAD4 activity, greater ROS production, and increased NETosis [28]. NETosis pathways observed in other cancers have also been implicated in NSCLC, including upregulation of IL-8 and HMGB1 activation [29].

#### 3.1.2. Clinical Data and Prognosis

NETs have also been associated with impaired CD8^+^ T cell function in patients with active NSCLC. Using serum MPO-DNA complexes to quantify NET burden, patients with high serum NETs consistently had lower densities of infiltrating CD8^+^ T cells in their tumors [30]. Lower neutrophil to CD8^+^ T cell ratios are also associated with longer progression-free intervals following PD-1 therapy, indicating this ratio could play a role in predicting clinical responses to PD-1 in NSCLC patients [30]. Retrospective analysis of tumor samples and clinical data from NSCLC patients has demonstrated a positive association between N2 neutrophils and NET levels within the TME [31]. Patients with high levels of NETs and N2 neutrophils had significantly decreased disease-free survival and overall survival [31]. This phenotype was also negatively associated with CD8^+^ T cell infiltration and positively associated with the number of anti-inflammatory Treg cells within the tumor, suggesting that NETs may play a role in decreasing the immune response to NSCLC [31].

#### 3.1.3. Treatment

Uncovering the role of NETs and their effect on immune cells provides new potential treatment avenues for NSCLC. A mouse model of NSCLC showed a significant decrease in tumor burden and restoration of CD8^+^-derived IFN-γ and IL-2 following treatment with A2AR inhibitors or CXCR2 inhibitors [25]. Given the compelling data that the N2 neutrophil phenotype is associated with increased NETosis and worse patient outcomes, therapies that have been shown to trigger a shift from N2 to N1 neutrophil subtypes such as TGF-β inhibitors are another promising avenue for further investigation [32].

### 3.2. Pancreatic Adenocarcinoma

#### 3.2.1. Preclinical Data

Neutrophils and NETs also appear to impact CD8^+^ T cell function in pancreatic ductal adenocarcinoma (PDAC). Work by Zhang et al. show that compared to healthy controls, both autochthonous and orthotopic mouse models of PDAC demonstrated significantly increased expression of IL-17, which attracts neutrophils to the tumor site and increases NET release [20]. Immunofluorescence showed CD8^+^ T cells were physically blocked from tumor entry where the NET meshwork was the densest, thus impairing their ability to kill tumor cells [20]. Blocking IL-17 resulted in decreased NETs in the TME, increased CD8^+^ T cell penetration into the tumor, and decreased markers of T cell exhaustion [20].

NETs also appear to enhance other established mechanisms of immune evasion in PDAC, including production of its characteristic dense stromal matrix and recruitment of myeloid-derived suppressor cells. NETs increase cancer-associated fibroblast activity and extracellular matrix deposition by activating pancreatic stellate cells, which impairs drug and immune cell infiltration [33]. Blocking NET formation in murine PDAC models decreases stromal buildup, slows tumor growth, and improves survival [33]. NETs may also form a positive feedback loop with cancer-associated fibroblasts, where each promotes the other’s activity, leading to increased fibrosis [33]. In addition, NETs contribute to immune suppression by recruiting myeloid-derived suppressor cells, which inhibit anti-tumor immune responses. Reducing NETs has been shown to decrease MDSCs and increase tumor-infiltrating T cells [33].

#### 3.2.2. Clinical Data

Serum IL-17 levels are also significantly higher in human patients with pancreatic cancer compared to healthy controls and may have some diagnostic value [34]. Analysis of tumor samples from patients with PDAC showed significantly shorter disease-specific survival and progression-free survival in patients with high neutrophil densities and NET levels [35]. This sample also demonstrated patients with low NET and neutrophil levels are more likely to respond to adjuvant chemotherapy [35]. These differences in prognosis and treatment response could be due to the inhibitory effects of NETs on CD8^+^ T cell cytotoxicity.

#### 3.2.3. Treatment

Il-17 is a promising target for PDAC treatment. Although anti-IL17 had little effect on tumor progression alone, anti-IL17/IL17R/PD-1 triple therapy significantly decreased tumor growth in a subcutaneous PDAC mouse model [20]. The therapeutic benefits of IL-17 inhibition were lost if CD8^+^ T cells were depleted, which confirms that the unmasking of the tumor to cytotoxic T cells is the potential primary therapeutic mechanism of IL-17 blockade [20].

### 3.3. Cholangiocarcinoma

#### 3.3.1. Preclinical Data

Increased CXCL6 expression is associated with higher levels of factors promoting cholangiocarcinoma cell growth, migration, and angiogenesis [36]. Depletion of CXCL6 in a mouse model of cholangiocarcinoma also demonstrated significantly decreased tumor size, fewer NETs in the TME, and increased CD8^+^ T cell tumor infiltration [36]. This mechanism is mediated by CXCL6 activating CXCR1/2 on neutrophils, which stimulates the RAS/MAPK pathway and induces NETosis [36].

#### 3.3.2. Clinical Data

ELISA analysis of serum from 26 cholangiocarcinoma patients prior to chemotherapy found a poor response to chemotherapy was associated with increased CXCL6 levels [36]. These patients also had a shorter overall survival and event-free survival compared to patients with lower levels of CXCL6 [36]. However, while this is a promising initial finding, it is important to note that interpretation of this dataset is constrained by its relatively small sample size and these clinical findings require validation in larger, multi-center, prospective validation cohorts. A larger study comparing the peripheral blood from 141 cholangiocarcinoma patients to 131 healthy controls found fewer circulating CD8^+^ T cells and more Tregs in the cholangiocarcinoma group [37]. Based on preclinical data around CXCL6, it is possible it plays a role in the development of these anti-inflammatory T cell profiles. Patients with positive NET staining within the tumor bed after resection also had significantly worse overall survival compared to patients with negative NET immunofluorescence, indicating a significant role of NETs (possibly induced by CXCL6) in this pro-cancer axis [38].

#### 3.3.3. Treatment

In vitro experiments knocking down CXCL6 demonstrated a synergistic treatment effect with gemcitabine, which is currently a first line treatment for cholangiocarcinoma [36,39]. CXCL6 knockdown also improved PD-1 therapy efficacy, further indicating it could be a promising target for cholangiocarcinoma therapy [36]. Together, these findings support a model in which CXCL6-driven NET formation facilitates tumor progression and resistance to therapy by creating an immunosuppressive TME that limits CD8^+^ T cell infiltration and function.

### 3.4. Colorectal Cancer

#### 3.4.1. Preclinical Data

It was recently demonstrated that mice subcutaneously injected with colorectal tumor cells have higher levels of NETs (measured by citrullinated histone H3 burden) compared to healthy controls [40]. These mice also demonstrated significant decreases in tumor volume when DNase I was added to PD-1 therapy to break down NETs compared to either therapy alone [40]. Dual treatment also increased CD8^+^ T cell tumor infiltration and cytotoxic activity compared to controls and either monotherapy [40]. These findings suggest that NETs play a significant role in resistance to treatment by dampening the CD8^+^ T cell response in colorectal cancer.

#### 3.4.2. Clinical Data and Prognosis

Analysis of tumor samples from patients with colorectal carcinoma show a negative correlation between PAD4 expression, which is strongly associated with NETosis and increased neutrophil infiltration, and overall survival [41]. Recent research has also found that increased presence of NETs in pre-treatment biopsies from patients with rectal cancer are significantly associated with a poor response to neoadjuvant chemotherapy [42]. These differences in chemotherapy responses could partly be due to exclusion of cytotoxic T cells from the tumor by NETs. Multiplex imaging showed that in tumors with high NET density, CD8^+^ T cells were often restricted to the surrounding stroma [42]. Based on these findings, NET density in rectal tumor biopsies could potentially serve as a biomarker that helps clinicians guide treatment selection and optimize therapeutic decision making.

#### 3.4.3. Treatment

Immune checkpoint inhibitors can be effective in solid tumors, including some colorectal cancers [41]. One example of this is through programmed cell death (PD-1) blockade, which prevents PD-1 from binding to PD-L1 on CD8^+^ T cells. By disrupting this inhibitory signaling pathway, immune checkpoint inhibitors restore T cell activation and enhance the immune system’s ability to recognize and eliminate tumor cells [43]. These treatments are efficacious in patients with mismatch repair deficient tumors, likely due to increased tumor mutational burden, antigen presentation, and immune cell infiltration [44]. The findings from Zhang et al. showing improved response to anti-PD-1 therapy in combination with DNase I treatment offers a promising avenue for overcoming treatment immunotherapy resistance in mismatch repair proficient colorectal tumors.

The glucagon-like peptide 1 (GLP-1) receptor agonist Exanitide may also enhance anti-PD-1 therapy by decreasing neutrophil free radical production and NET release [45]. Additionally, mice that had their tumors cleared by the Exenatide/anti-PD-1 combination were able to later reject new tumor cells, suggesting the treatment successfully activated long-term immune memory [45]. Glycyrrhizic acid is a potent inhibitor of PAD4 and reduces levels of neutrophil chemoattractants IL-1β, IL-6, and TNF-α in colorectal cell cultures [46]. Glycyrrhizic acid also increased surveillance by CD8^+^ T cells and slowed progression of polyps in a mouse model of colitis-associated colorectal cancer [46]. Another potential adjunctive therapy is iron chelation. Iron is necessary for the oxidative burst, which produces radical oxygen species that trigger NET release. Recent research shows that introduction of an iron chelating nanoplatform into the colorectal TME can prevent the formation of NETs and increase CD8^+^ T cell tumor infiltration [47]. Anti-PD-L1 therapy, which failed to decrease tumor size as a monotherapy, became effective when combined with the iron-regulating nanoparticles [47]. Together, these findings indicate that NET density could serve as a datapoint for determining which patients are more likely to respond to immunotherapy and which patients may benefit from combination therapies.

### 3.5. Bladder Cancer

#### 3.5.1. Preclinical Data

Preclinical data suggests that NETs modulate the immune response in multiple ways in bladder cancer. NETs increase the hormone Stanniocalcin-1 in a mouse model of metastatic bladder cancer [48]. In previous research, Stanniocalcin-1 has been shown to suppress CD8^+^ T cell recognition and cytotoxic killing of cancer cells [49]. A separate mouse model found that mice treated with radiation therapy had more NETs in the TME and NET inhibition significantly improved response to radiation [50]. Similar to several other cancers, this mechanism of NETosis appears to be PAD4-dependent [50]. These findings are significant because radiotherapy is often thought to make tumors more susceptible to immune infiltration, but if NETs are present, they may counteract this effect by excluding CD8^+^ T cells from the tumor interior [50].

#### 3.5.2. Clinical Data and Prognosis

Immunofluorescence analysis of tumor tissue samples from 71 patients with bladder cancer compared to healthy controls demonstrated increased NETosis and decreased serum Dnase l activity, which could be restored in vitro with recombinant human Dnase I [51]. Additionally, a high neutrophil to CD8^+^ T cell ratio is linked to worse prognosis in bladder cancer patients [50]. Finally, recent studies have isolated several genes associated with differential NETosis in bladder cancer patients, and those linked to increased NETosis were in turn associated with worse prognosis and decreased treatment sensitivity [52].

#### 3.5.3. Treatment

Contrary to many of the other cancer types discussed, a specific subset of NETs associated with Bacillus Calmette–Guerin (BCG) treatment may play a protective role in bladder cancer. BCG, a live attenuated form of *Mycobacterium bovis* traditionally used as a tuberculosis vaccine, is an effective treatment for non-muscle invasive bladder cancer when administered intravesically via catheter [53]. Although its mechanism of action is not fully understood, emerging evidence suggests that one pathway involves the induction of NETosis, which in this context appears to promote cell-cycle arrest and inhibit tumor growth [54]. This phenomenon introduces an apparent mechanistic paradox, contrasting with the broader narrative of this review that NETs promote tumorigenesis. One possible explanation for this contradiction is the difference between acute, pathogen-induced inflammation and chronic, tumor-induced immunosuppression. Unlike the structural, immunosuppressive NET scaffolds induced by chronic inflammation as cancer progresses, intravesical BCG instillation triggers an acute, highly localized increase in immune activity [54]. In this specific therapeutic context, the resulting BCG-induced NETs serve as localized inflammatory adjuvants that physically entrap and concentrate immunostimulatory microbial antigens, promoting cell-cycle arrest and facilitating a robust acute immune response that destroys superficial tumor cells before a chronic, suppressive microenvironment can be established.

Importantly, patients with advanced muscle-invasive bladder cancer are not eligible for treatment with BCG and may benefit more from treatments that inhibit NETs based on preclinical and clinical findings that NETs can be associated with worse prognosis and impaired radiation therapy efficacy. For these patients, degrading NETs with DNase I to enable CD8^+^ T cell tumor infiltration is a potential adjunctive treatment approach. Nevertheless, further investigation is needed to fully evaluate the therapeutic efficacy of NET-directed interventions in bladder cancer.

### 3.6. Hepatocellular Carcinoma

#### 3.6.1. Preclinical Data

Like many other cancer types discussed previously, NETs have been shown to physically exclude CD8^+^ T cells from the HCC tumor interior, limiting their cytotoxic efficacy [7]. One proposed mechanism for NETosis in hepatocellular carcinoma (HCC) is increased production of IL-8 by hepatocytes, which induces NETosis by binding CXCR1 and 2 on neutrophils [7]. Hepatic stellate cells and liver sinusoidal endothelial cells also produce TGF-β in HCC and other inflammatory states [55]. TGF-β in the TME induces NETosis in HCC and can recruit CD8^+^ T cells. This in turn induces CD8^+^ T cell production of TGF-β, creating a positive feedback loop [56]. This mechanism appears to be mediated by blocking the nuclear translocation of NF-κB p65 through NETs binding to the transmembrane and coiled-coil domain 6 (TMCO6) on T cells, which shuts down a pathway essential for T cell activation and survival [56]. These T cells then produce high levels of exhaustion markers PD-1 and Tim-3 and fewer cytotoxic cytokines [56]. The significance of TMCO6 was confirmed by experiments showing Hepa1-6 tumors grown in TMCO6-deficient mice grew much slower than in wild type mice and CD8^+^ T cells remained highly active even in the presence of NETs in this model [56]. While these findings delineate a compelling and novel mechanistic axis, it must be emphasized that the TMCO6-NET interaction relies almost entirely on a single seminal study. These findings have not yet been independently validated across diverse experimental models or separate institutional cohorts, meaning further cross-validation is required before TMCO6 can be definitively classified as a driver of hepatic T cell dysfunction.

#### 3.6.2. Clinical Data and Prognosis

Clinical data further support the importance of NETs in HCC. In CD8^+^ T cells isolated from HCC patients, siRNA-mediated knockdown of TMCO6 prevented NET-induced CD8^+^ T cell exhaustion, as reflected by preserved IFN-γ and granzyme B production [56]. In addition, neutrophils from HCC patients, particularly those with metastatic disease, exhibit an increased capacity for NET formation compared to healthy controls [57].

Consistent with these findings, the presence of NETs within HCC tumor tissues is associated with worse prognosis [58]. Increased levels of NET-associated cathepsin G in tumor tissue is also linked to worse clinical outcomes and implicated in HCC metastases in both in vivo and in vitro models [58]. Elevated circulating IL-8 levels, a factor associated with NET formation in HCC, correlates with reduced response to immune checkpoint blockade [59]. Finally, a set of six NET-related genes were identified using patient data from the Cancer Genome Atlas-Liver Hepatocellular Carcinoma that can predict survival outcomes in patients with HCC, indicating there may be a role for genetic testing in HCC prognosis [60]. However, such genetic prognostic signatures carry notable methodological limitations that warrant caution. Transcriptomic models derived from bulk tissue databases are highly susceptible to algorithmic overfitting and retrospective cohort bias. Furthermore, these signatures frequently lack extensive external validation in independent clinical datasets that account for the diverse underlying etiologies of HCC, such as distinguishing between hepatitis B, hepatitis C, or non-alcoholic steatohepatitis-driven malignancies, which could alter the background immune landscape.

#### 3.6.3. Treatment

In addition to its observed role in HCC progression, TMCO6 could also be a promising therapeutic target. Combining the inhibition of the NET-TMCO6 axis with anti-PD-1 therapy showed significant improvement in tumor shrinkage compared to PD-1 inhibitors or DNase I alone in a mouse model of HCC [56]. The IL-8/CXCR2 interaction is another promising therapeutic target. An orthotropic mouse model of HCC was sensitized to PD-1 therapy and demonstrated decreased tumor growth and prolonged survival after treatment with a CXCR2 inhibitor [59]. This pathway could be a therapeutic target for patients previously unresponsive to immunotherapy. The GLP-1 agonist Liraglutide similarly shows promise as an adjunctive treatment. Work by Chen et al. demonstrated that Liraglutide binds GLP-1 receptors on neutrophils, which triggers a signaling cascade that suppresses the NADPH oxidase activity and ultimately decreases free radical production and PAD4 expression in a mouse model of liver cancer [61]. Liraglutide reduced NET markers (CitH3 and MPO) within the tumor tissue and in the peripheral blood, significantly increased the density of CD8^+^ T cells inside the tumor core, and increased CD8^+^ T cell IFN-γ and TNF-α release [61]. While PD-1 had little effect on these tumors on its own, adding Liraglutide resulted in significantly decreased tumor burden [61]. These findings invite further investigation into whether Liraglutide could be an effective combination treatment in human patients with HCC.

Targeting NETs could also offer a potential opportunity to reduce tumor recurrence. Inhibiting NETs with DNase I or PAD4 inhibitors decreases HCC metastasis in mice [62]. Combining DNase I with anti-inflammatory drugs (i.e., aspirin and hydroxychloroquine) has a synergistic effect that further reduces HCC metastasis in mice [57]. Another mouse model expanded on these findings to show that removing NETs with DNase I restored CD8^+^ T cell function and decreased HCC tumor recurrence [63]. Cheng et al. also demonstrated that neutralizing the acidity of the TME with a pH responsive hydrogel in combination with DNase I significantly increases CD8^+^ T cell efficacy and prevents HCC recurrence in mice when administered with NK cell infusions [63]. These findings invite further investigation into the untapped therapeutic value of NET blockade and degradation not only for treatment, but also potentially for recurrence prevention.

### 3.7. Skin Cancer

NETs appear to play different roles in non-melanoma skin cancer (NMSC) and melanoma. Immunofluorescence staining of tumor tissue samples from 53 patients with NMSC revealed a positive correlation between ulceration diameter, cross-sectional area, and neutrophil invasion [64]. Interestingly, unlike in many other cancer types, the presence of neutrophils or NETs were not associated with reduced CD8^+^ T cell infiltration [64]. On the other hand, NETs appear to be associated with more severe phenotypes in melanoma. Analysis of metastases from melanoma patients found that about half contained neutrophils and slightly less than one third contained NETs, but all of the metastases greater than 2.1 cm contained NETs [65]. Further, NETs were associated with tumor necrosis, which corresponds with worse outcomes in cutaneous melanoma [65,66]. Serum NET levels have also been shown to correlate inversely with density of CD8^+^ T cells in tumor samples from patients with metastatic melanoma [14]. A recent study of mixed tumor samples including melanoma has also demonstrated that dendritic cell-specific DNASE1L3, which degrades NETs, promotes CD8^+^ T cell tumor infiltration, reduces exhaustion, and enhances anti-PD-L1 therapy efficacy [67].

Several biological mechanisms may explain the differing roles of NETs in NMSC and melanoma. In NMSCs, the tumor microenvironment is dominated by chronic, localized inflammation induced by UV damage. In this setting, neutrophil recruitment and NET deposition seem to function primarily as a mechanical response to tissue remodeling and wound healing at sites of surface ulceration. Because this inflammatory process remains highly localized, it may not have a significant enough impact on systemic chemokine gradients to impact CD8^+^ T cell migration [64]. NMSCs also rarely metastasize or aggressively invade local tissues, so they may not reach a size at which NETs significantly inhibit CD8^+^ T cell infiltration to the tumor core. In contrast, melanoma can behave as a highly dynamic, vascularized malignancy that secretes potent systemic immunosuppressive and angiogenic factors like IL-8 and VEGF [68]. These drivers can recruit dense networks of pro-tumor N2 neutrophils, leading to the creation of more pronounced NET structural networks to shield melanoma cells from the adaptive immune system, explaining the strong correlation between NET density, structural tumor necrosis, and T cell exclusion observed uniquely in advanced melanoma [65].

### 3.8. Penile Cancer

While there is limited data on NETs in penile squamous cell carcinoma (SCC), research on the immune cell profiles in this cancer suggest more investigation into the presence and role of NETs may be warranted. Analysis of preoperative tumor samples from patients with penile SCC found that a high neutrophil to lymphocyte ratio was associated with higher NET levels, increased density of exhausted CD8^+^ T cells, as measured by markers such as PD-1, Tim-3, and Lag-3, and greater resistance to PD-1 inhibitors and cisplatin chemotherapy [69]. High neutrophil to lymphocyte ratio was also an independent indicator of progression-free survival and overall survival in these patients [69]. Another study found tumor samples from patients with squamous cell penile cancer had significantly lower numbers of CD8^+^ effector T cells compared to peripheral blood samples from the same patients [70]. These preliminary findings associating NETs with poorer outcomes and characterizing the T cell environment invite further investigation into the efficacy of adjunctive therapies targeting NETs to restore CD8^+^ T cell cytotoxic function in penile SCC.

## 4. Conclusions

**Figure 2:**
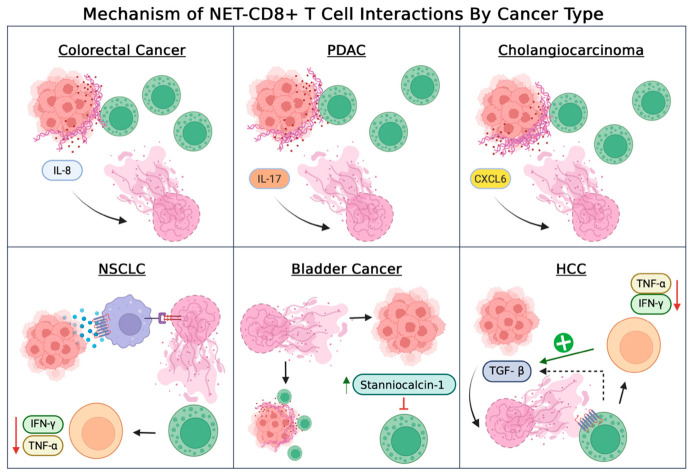
In colorectal cancer, IL-8-induced NETs form barriers that prevent CD8^+^ T cell infiltration into the tumor core. In pancreatic ductal adenocarcinoma (PDAC), IL-17 recruits neutrophils and enhances NET formation, physically excluding CD8^+^ T cells from tumors and impairing cytotoxic function. In cholangiocarcinoma, CXCL6 activates CXCR1/2 and the RAS/MAPK pathway to drive NETosis, which decreases CD8^+^ T cell infiltration. In non-small cell lung cancer (NSCLC), A2AR-driven CXCL5 on macrophages induces NETosis by binding CXCR2 on neutrophils. These NETs modulate cGAS-STING signaling in CD8^+^ T cells and induce exhaustion and reduced effector cytokine production. In bladder cancer, NETs upregulate stanniocalcin-1, suppressing CD8^+^ T cell recognition and cytotoxicity. NETs also physically exclude CD8^+^ T cells from the tumor interior. IL-8 and TGF-β drive NETosis, which increases CD8^+^ T cell exhaustion. These exhausted T cells produce fewer cytotoxic cytokines and produce more TGF-β, creating a positive feedback loop. Created in BioRender. Ruff, S. (https://BioRender.com/dbos6ls, accessed on 22 June 2026) is licensed under CC BY 4.0.

NETs are important modulators of immune activity in cancer, particularly of cytotoxic CD8^+^ T cells. They have been shown to physically exclude CD8^+^ T cells from the tumor interior, contribute to their exhaustion, and impair their cytotoxic effects (Figure 2). As these interactions and their mechanisms come to light, they offer insight into new prognostic and treatment avenues that may offer efficacy in tumors resistant to current therapies. While further research is needed to extend many of these preclinical findings to clinical trials, targeting the NET-CD8^+^ axis shows promise for improving outcomes in many types of cancer.

### Future Directions

Despite growing evidence that NETs suppress CD8^+^ T cell-mediated anti-tumor immunity, major gaps remain in our understanding of the context-specific mechanisms governing this interaction. Future work should prioritize dissecting the molecular determinants of NET-driven T cell dysfunction, including the relative contributions of physical immune exclusion, checkpoint signaling, and metabolic suppression. Furthermore, integrating preclinical discoveries with biomarker-driven clinical studies will be critical for determining how NET-targeted approaches can be rationally combined with current cancer therapies. Addressing these questions may help establish the NET/CD8^+^ T cell axis as both a prognostic framework and a therapeutic opportunity in oncology.

## Data Availability

No new data were generated or analyzed in the preparation of this review. Therefore, data sharing is not applicable to this article.

## Abbreviations

The following abbreviations are used in this manuscript:

BCG	Bacillus Calmette–Guerin
CitH3	Citrullinated histone H3
G-CSF	Granulocyte colony-stimulating factor
GLP-1	Glucagon-like peptide 1
GM-CSF	Granulocyte–macrophage colony-stimulating factor
HCC	Hepatocellular carcinoma
HMGB1	High mobility group box 1
MPO	Myeloperoxidase
NETs	Neutrophil extracellular traps
NMSC	Non-melanoma skin cancer
NSCLC	Non-small cell lung cancer
PAD4	Peptidyl arginine deiminase 4
PD-L1	Programmed death ligand 1
PDAC	Pancreatic ductal adenocarcinoma
ROS	Reactive oxygen species
SCC	Squamous cell carcinoma
TMCO6	Transmembrane and coiled-coil domain 6
TME	Tumor microenvironment
